# Research trends in cardiovascular tissue engineering from 1992 to 2022: a bibliometric analysis

**DOI:** 10.3389/fcvm.2023.1208227

**Published:** 2023-08-01

**Authors:** Ping Lai, Ming Sheng, Jin-hua Ye, Zhi-xian Tang, Shuo Hu, Bei Wang, Jing-lin Yuan, Yi-hong Yang, Yi-ming Zhong, Yong-ling Liao

**Affiliations:** ^1^ Department of Cardiology, First Affiliated Hospital of Gannan Medical University, Gannan Medical University, Ganzhou, China; ^2^ Key Laboratory of Prevention and Treatment of Cardiovascular and Cerebrovascular Diseases, Ministry of Education, Gannan Medical University, Ganzhou, China; ^3^ Department of Library, Gannan Medical University, Ganzhou, China; ^4^ Department of Physiology, School of Basic Medical Sciences, Gannan Medical University, Ganzhou, China; ^5^ Department of Thoracic Surgery, First Affiliated Hospital of Gannan Medical University, Ganzhou, China; ^6^ Department of Heart Medical Centre, First Affiliated Hospital of Gannan Medical University, Ganzhou, China

**Keywords:** cardiovascular tissue engineering, potential research direction, biomaterial, bibliometric analysis, VOSviewer

## Abstract

**Background:**

Cardiovascular tissue engineering (CTE) is a promising technique to treat incurable cardiovascular diseases, such as myocardial infarction and ischemic cardiomyopathy. Plenty of studies related to CTE have been published in the last 30 years. However, an analysis of the research status, trends, and potential directions in this field is still lacking. The present study applies a bibliometric analysis to reveal CTE research trends and potential directions.

**Methods:**

On 5 August 2022, research articles and review papers on CTE were searched from the Web of Science Core Collection with inclusion and exclusion criteria. Publication trends, research directions, and visual maps in this field were obtained using Excel (Microsoft 2009), VOSviewer, and Citespace software.

**Results:**

A total of 2,273 documents from 1992 to 2022 were included in the final analysis. Publications on CTE showed an upward trend from 1992 [number of publications (Np):1] to 2021 (Np:165). The United States (Np: 916, number of citations: 152,377, H-index: 124) contributed the most publications and citations in this field. Research on CTE has a wide distribution of disciplines, led by engineering (Np: 788, number of citations: 40,563, H-index: 105). “Functional maturation” [red cluster, average published year (APY): 2018.63, 30 times], “cell-derived cardiomyocytes” (red cluster, APY: 2018.43, 46 times), “composite scaffolds” (green cluster, APY: 2018.54, 41 times), and “maturation” (red cluster, APY: 2018.17, 84 times) are the main emerging keywords in this area.

**Conclusion:**

Research on CTE is a hot research topic. The United States is a dominant player in CTE research. Interdisciplinary collaboration has played a critical role in the progress of CTE. Studies on functional maturation and the development of novel biologically relevant materials and related applications will be the potential research directions in this field.

## Introduction

Cardiovascular diseases, including myocardial infarction, ischemic cardiomyopathy, and heart failure, are caused by malfunctioning valves, blockage of blood vessels, or damaged heart muscle, and are the leading cause of death globally (1). Recent decades have seen significant advances in the treatment of these diseases (2, 3). However, novel therapies are required for regenerating the cardiomyocytes or tissues in the diseased heart tissues of patients with myocardial infarction or heart valve diseases (4).

Stem cells, such as induced pluripotent stem cells (iPSCs) (5) and embryonic stem cells (ESCs) (6), are characterized by their ability to self-renew and differentiate into various cell types in the human body (7), including cardiomyocytes (8). Currently, stem cells are increasingly being used in the field of regenerative medicine (9), especially in cardiovascular diseases (10). Several methods have been developed for delivering stem cells to the injury site to replace the lost cardiomyocytes in patients with cardiovascular diseases (11). However, the direct delivery of stem cells has not been successful because the percentage of the delivered stem cells differentiating into functional cardiomyocytes was significantly low and several cases showed the formation of teratomas (12, 13). Therefore, to overcome these issues, stem cells were first *in vitro* differentiated into cardiomyocytes and then transferred to the impaired site (14). However, the results were unsatisfactory because of a high rate of cell death and a limited number of viable cardiomyocytes after in-vitro differentiation (15).

The emergence of tissue engineering has further highlighted the clinical application of stem cells. For example, the survival of cardiomyocytes was significantly increased by attachment to a suitable supporting surface (16). The goal of tissue engineering is to assemble functional scaffolds with biologically acceptable materials, which provide a viable cellular environment for restoring, maintaining, or improving damaged tissues or whole organs (17). Tissue engineering takes into account the 3-dimensional (3D) structure of the tissue and combines the scaffolding biomaterials with the stem cells to gradually develop functional tissues, either completely or partly (18). Cardiovascular tissue engineering (CTE) involves the generation of specific high-differentiating cardiac tissues including heart muscles, valves, or vessels for the study and treatment of cardiovascular disease (19, 20). In the last three decades, the development of CTE has been rapid, including studies on novel biomaterials (21), mechanisms of cardiovascular disease (22), and the recovery of heart function (23).

Bibliometrics is the statistical analysis of publications in the area of interest and includes quantitative analysis of the literature regarding the characteristics of the publications, author output and impact, keywords, and references (24). Bibliometrics is widely used in many fields of medicine, including surgery (25), internal medicine (26), signaling pathways (27), and stem cell research (28).

In this study, we performed bibliometric analysis to determine the current status and challenges, evolutionary path, research hotspots, and future research direction in the area of CTE.

## Methods

### Search strategy

**Figure 1:**
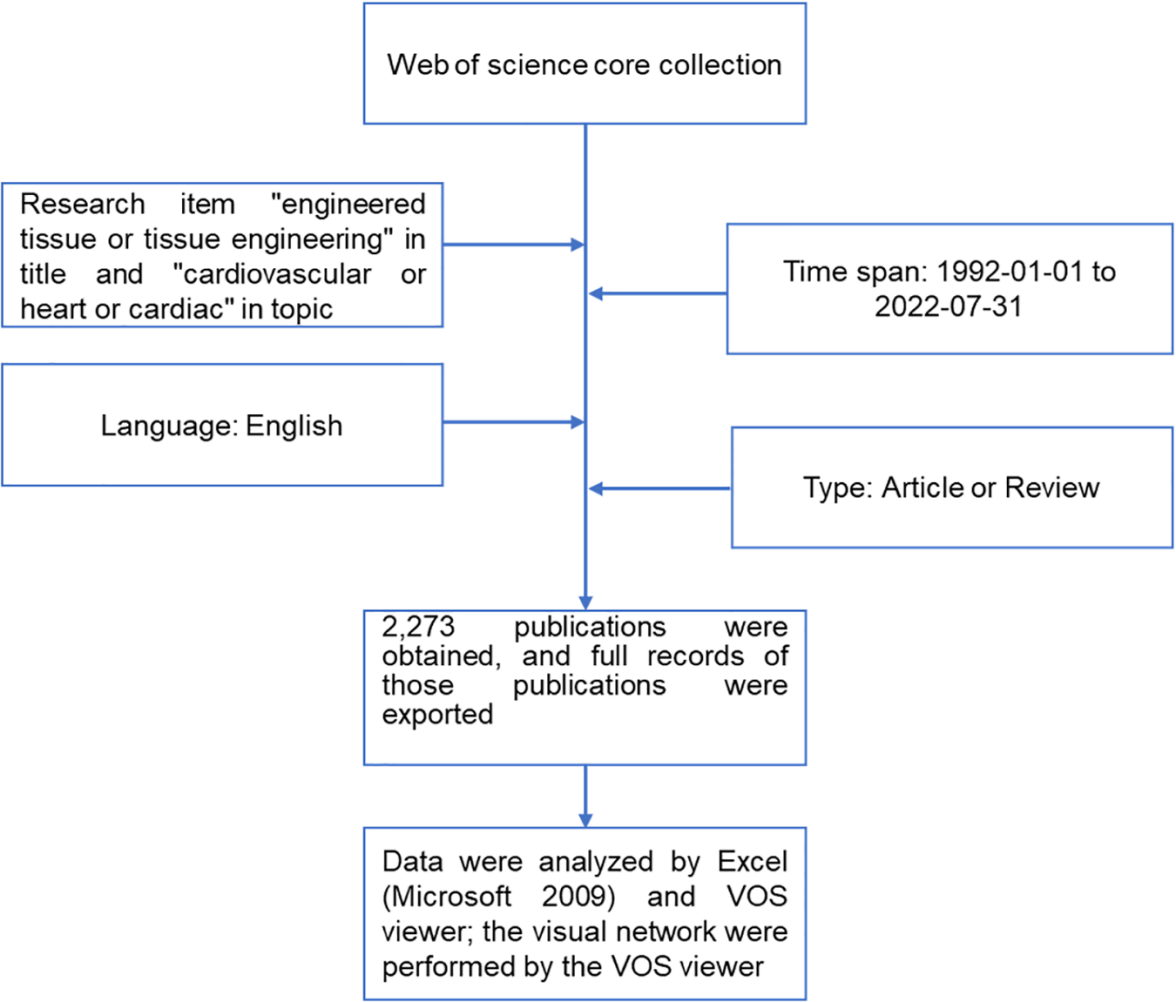
The flow chart of this bibliometric analysis.

The Web of Science Core Collection (WoSCC) was used as the data source for our research. WoSCC is the world's leading citation database with the most comprehensive collection of articles from high-impact journals, open-source journals, conference meetings, and books. Therefore, we performed the following search in the WoSCC database on 5 August 2022: TI = “engineered tissue or tissue engineering” AND TS = “cardiovascular or heart or cardiac”; dates, “1992-01-01 to 2022-07-31”. Only articles and reviews that were published in English were extracted. The search records, including names of all the authors, titles of the articles or reviews, time of publication, journal title, affiliations, keywords, citations, and references were exported as plain text and Excel files. The flow chart of the bibliometric analysis is shown in Figure 1.

### Data analysis

Microsoft Excel 2009 was used to analyze the citations over time (each year) and the distribution of countries or regions, top authors, research institutions, research areas, and journals. The number of publications (Np) was calculated for all authors, countries, journals, and other specified parameters. The number of citations without self-citations (Nc) and the average citation number (ACN; total citations/total publications) were used for estimating the degree of influence (high or low) of the author or the journal. The h-index from WoSCC was used to assess the scholarly contributions of the researchers and forecast the future scientific achievements of the investigators, countries, or institutions. The h-index was also used to determine the influence of the journal's publications. The main bibliometric indicators included the total number of publications, main authors (including institutions and countries), and citations. VOSviewer (v.1.6.18, CWTS, Leiden University) was used to visualize the network of co-citations, authors, countries, and keywords (without the search items). JAVA-based VOSviewer and CiteSpace (version 6.1.R3) tools were used to analyze the clusters of keywords from publications with high citation bursts. CiteSpace parameters were as follows: period (1992–2022); years per slice (one year); term source (title, abstract, author keyword, and keyword plus); node types (keyword); links (strength: cosine, scope: within slices); selection criteria (g-index: k = 25); and pruning (minimum spanning tree, pruning sliced networks, and pruning the merged network).

## Result

### Publications and citations

**Figure 2:**
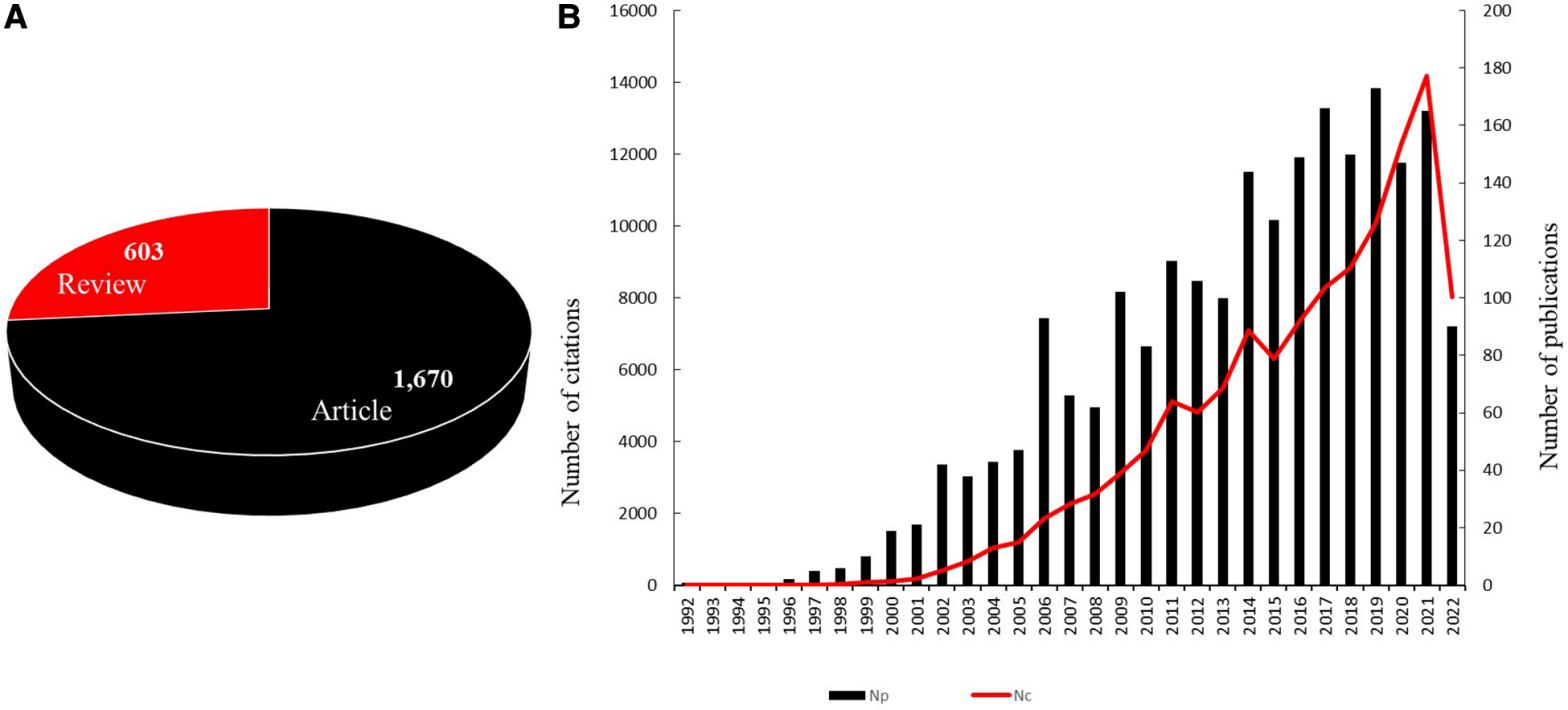
(**A**) Type of publications included in the current study. (**B**) The trend of publications and citations globally in the CTE research area between 1992 and 2022.

The final bibliometric analysis included 2,273 publications, of which 1,670 were articles and 603 were reviews. The total number of publications on CTE per year showed an upward trend from 1992 (Np:1) to 2021 (Np:165). Since 2009, more than 100 publications were published on CTE every year except in 2010 (Np:83). The highest number of publications on CTE was published in 2019 (Np:173). The citations of all the publications on CTE matched the trend of the publications on CTE. The total number of citations on CTE increased significantly after 2015 and peaked in 2021 with 14,189 citations (Figures 2A,B).

### Countries/regions distribution

The United States (Np:916), Germany (Np:315), and China (Np:223) were the top three countries with the highest number of citations in the field of CTE. The top three countries accounted for 1,454 (63.97%) publications out of the 2,273 publications analyzed. The top three countries for the ACN index were the USA, China, and Germany (Table 1). This indicated robust research regarding CTE in these three countries.

**Table 1:** Ten countries with the most CTE-related papers.

Country	Np	Nc	H-index	ACN
USA	916	52,377	124	57.18
Germany	315	14,044	64	44.58
China	223	6,606	40	29.62
Canada	149	9,909	49	66.50
Netherlands	144	5,562	43	38.63
England	140	6,140	40	43.86
Japan	124	8,167	42	65.86
Italy	108	3,174	35	29.39
Switzerland	96	5,294	41	55.15
Iran	89	2,583	27	29.02

Np, The number of papers; Nc, The number of citations excludes self-citations; ACN, average citation number; and APY, Average published year.

**Figure 3:**
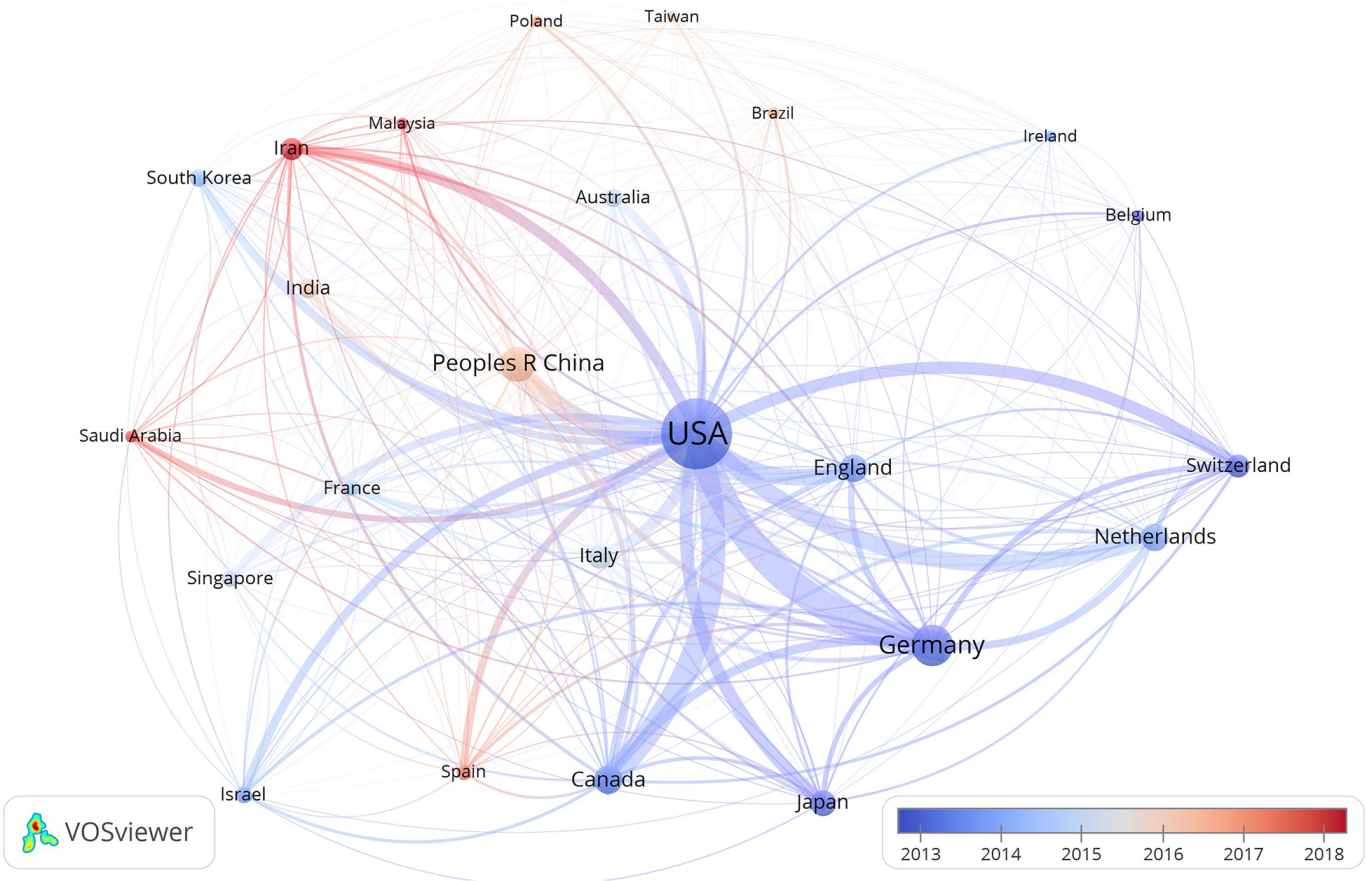
Map of countries/regions with more than 20 publications on the topic of CTE. The node size indicates the number of papers; the color of the node indicates the year in which the country/region started research in CTE, with blue denoting early, and red denoting late; cooperation between the countries/regions is represented by a line.

Figure 3 shows the map of countries/regions with more than 20 publications over time. The highest number of publications were from research labs in the USA. Furthermore, in the early period, the USA and Germany led the research regarding CTE, whereas China and Iran are emerging countries in this area. Furthermore, researchers from the USA collaborated significantly with researchers from South Korea, Japan, Canada, and Australia; researchers from Germany collaborated closely with researchers from England, the Netherlands, France, Switzerland, and Spain; researchers from China collaborated with researchers from Singapore, Malaysia, Poland, and Spain (Figure 3).

### Top ten authors and research institutions

**Figure 4:**
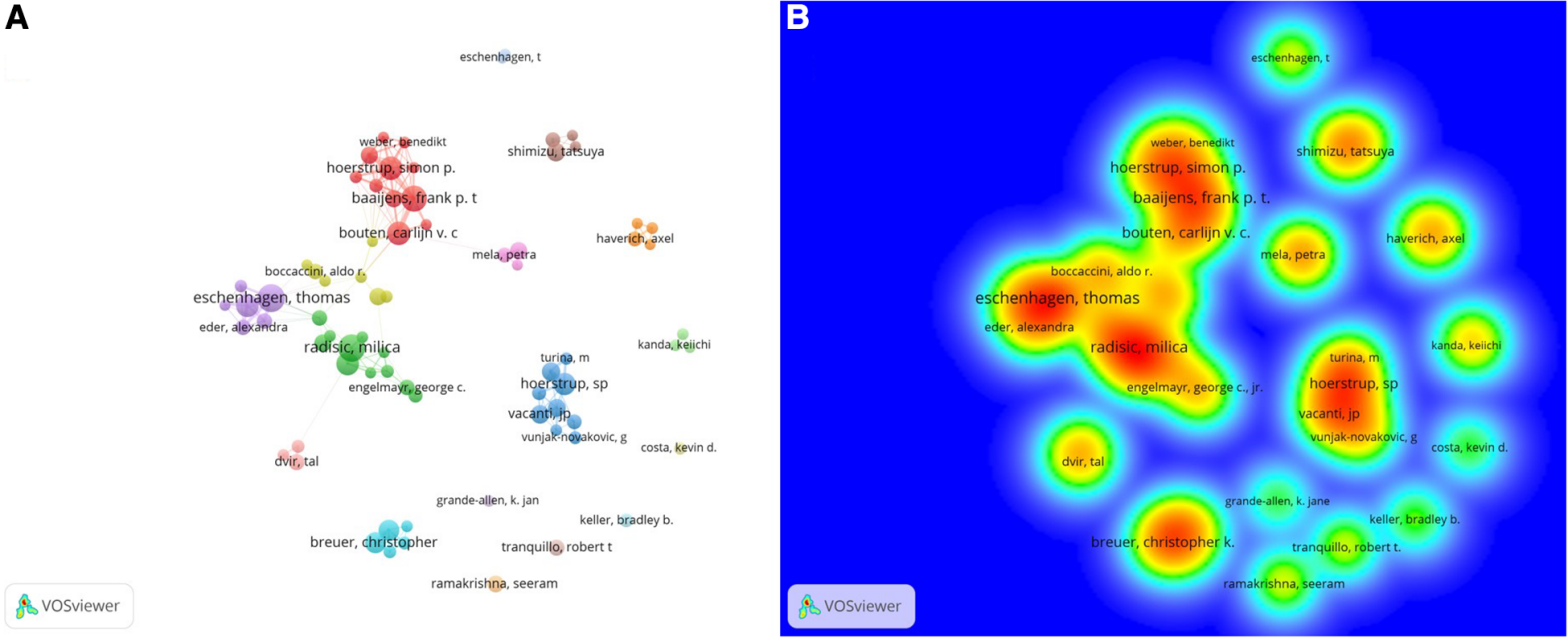
(**A**) Visual network of co-authors in CTE research. Each node represents an author; authors with close relationships are allocated to one cluster with a specific color. (**B**) The density visualization map shows the co-citation relationship between authors and co-authors.

Among the top ten productive authors, Hoerstrup SP (Np:75) from Switzerland ranked first, followed by Eschenhagen T (Np:65) from Germany and Radisic M (Np:58) from Canada. Furthermore, four of the top ten productive authors were from the USA, and two each were from Germany and the Netherlands (Table 2). Eschenhagen T worked closely with Eder A, and Hoerstrup SP collaborated with Vacanti JP and Turina M (Figures 4A,B).

**Table 2:** Top ten of the most fruitful authors and institutions.

Author	Np	Nc	H-index	Country/region	ACN
Hoerstrup SP	75	4,912	41	Switzerland	65.49
Eschenhagen T	65	5,677	33	Germany	87.34
Radisic M	58	4,405	34	Canada	75.95
Baaijens FPT	57	2,603	28	Netherlands	45.67
Vunjak-novakovic G	50	5,911	31	USA	118.22
Bouten CVC	47	1,878	23	Netherlands	39.96
Shinoka T	41	2,543	24	USA	62.02
Breuer CK	37	1,524	21	USA	41.19
Hansen A	37	2,256	21	Germany	60.97
Mayer JE	36	3,533	31	USA	98.14
Institution					
Harvard university	154	16,367	74	USA	106.28
Eindhoven university of technology	93	3,173	33	Netherlands	34.12
University of Toronto	79	6,593	39	Canada	83.46
Massachusetts institute of technology	77	9,638	50	USA	125.17
University of hamburg	71	4,837	32	Germany	68.13
University of Zurich	70	4,201	37	Switzerland	60.01
German centre for cardiovascular research	68	2,453	27	Germany	36.07
University medical center hamburg eppendorf	67	4,073	30	Germany	60.79
Pennsylvania commonwealth system of higher education	59	3,361	33	USA	56.97
University of california system	55	3,084	26	USA	56.07

Np, The number of papers; Nc, The number of citations excludes self-citations; ACN, average citation number; and APY, Average published year.

Harvard University was the top research institution with 154 publications, followed by the Eindhoven University of Technology with 93 publications and the University of Toronto with 79 publications. The Massachusetts Institute of Technology (MIT) from the USA showed the highest ACN (125.17), thereby suggesting a significant influence of the USA in CTE research (Table 2).

### Top ten research areas and journals

The top three research disciplines with the highest number of publications were *engineering* (Np: 788, Nc: 40,563, H-index: 105), *materials science* (Np: 752, Nc:41,762, H-index: 105), and *cell biology* (Np: 539, Nc: 23,259, H-index: 79) (Table 3).

**Table 3:** Top ten research areas.

Research areas	Np	Nc	H-index
Engineering	788	40,563	105
Materials science	752	41,762	105
Cell biology	539	23,259	79
Cardiovascular system cardiology	353	19,081	78
Science technology other topics	240	10,550	56
Biotechnology applied Microbiology	208	10,531	55
Chemistry	165	9,236	49
Biochemistry molecular biology	164	9,168	49
Surgery	131	7,841	50
Research experimental medicine	127	5,111	33

Np, The number of papers and Nc, The number of citations excludes self-citations.

Among the top ten journals with the greatest number of publications, *Tissue Engineering Part A* (Np: 84, Nc: 3,049, ACN: 36.30) ranked first and was followed by *Biomaterials* (Np: 82, Nc:14,138, ACN: 172.41) and *Acta Bio-material* (Np: 64, Nc:4,156, ACN: 64.94). Furthermore, three of the top ten journals, namely, *Biomaterials*, *Acta Biomaterial*, and *Circulation,* were high-impact factor journals with an impact factor ≥10.0 (Table 4). *Biomaterials* showed the highest ACN value of 172.41 (Table 4).

**Table 4:** Top ten fruitful journals.

Journal	Np	Nc	H-index	IF	ACN
Tissue engineering part A	84	3,049	32	4.08	36.30
Biomaterials	82	14,138	60	15.304	172.41
Acta bioMaterialia	64	4,156	37	10.633	64.94
Tissue engineering	52	4,926	36	NA	94.73
Tissue engineering part C: Methods	43	1,115	18	3.273	25.93
Journal of tissue engineering and regenerative medicine	33	1,251	18	4.323	37.91
Annals of biomedical engineering	32	1,309	17	4.219	40.91
Tissue engineering part B: Reviews	32	3,106	20	7.376	97.06
Journal of biomedical materials research part A	30	1,963	20	4.854	65.43
Circulation	29	3,957	26	39.918	136.45

Np, The number of papers; Nc, The number of citations excluding self-citations; IF, impact factor; ACN, average cited number; and NA, not applicable.

### Highly co-citation publications

**Figure 5:**
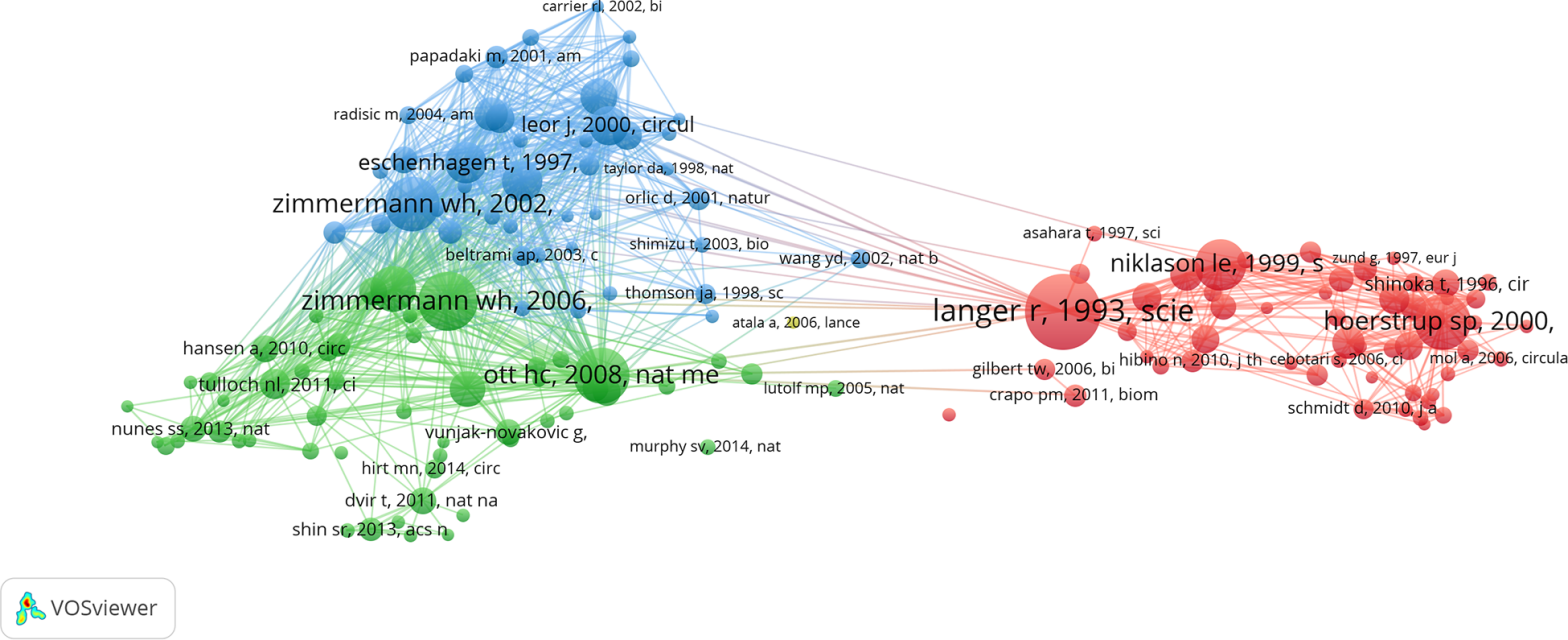
Map of the most co-cited publications (more than 50 times). The node size denotes the number of citations, with a larger node representing a higher number of citations. The clusters are represented by different colors. The lines connecting different nodes show the relationship between different publications.

Among the top ten papers with the most co-citations, nine were articles and one was a review. Seven of these were published after the year 2000. The earliest article was published in 1993 and the most recent article among the top ten was published in 2008. Zimmermann WH was the only author with two publications among the top ten most co-cited papers. *Circulation*, *Science*, and *Nature Medicine* had two articles each featuring among the top ten most co-cited papers (Table 5). Furthermore, 151 papers had more than 50 co-citations (Figure 5).

**Table 5:** Top ten most co-cited publications.

Rank	Author	Title	Type	Year	Doi	Journal	Citations	Total link strength
1	Langer R, et al.	Tissue engineering	Review	1993	10.1126/science.8493529	Science	297	2,194
2	Zimmermann WH, et al.	Engineered heart tissue grafts improve systolic and diastolic function in infarcted rat hearts	Article	2006	10.1038/nm1394	Nature medicine	234	3,265
3	Ott HC, et al.	Perfusion-decellularized matrix: using nature's platform to engineer a bioartificial heart	Article	2008	10.1038/nm1684	Nature medicine	221	2,719
4	Hoerstrup SP, et al.	Functional living trileaflet heart valves grown *in vitro*	Article	2000	10.1161/01.cir.102.suppl_3.iii-44	Circulation	217	1,919
5	Zimmermann WH, et al.	Tissue engineering of a differentiated cardiac muscle construct	Article	2002	10.1161/hh0202.103644	Circulation research	214	3,197
6	Niklason LE, et al.	Functional arteries grown *in vitro*	Article	1999	10.1126/science.284.5413.489	Science	202	1,715
7	Radisic M, et al.	Functional assembly of engineered myocardium by electrical stimulation of cardiac myocytes cultured on scaffolds	Article	2004	10.1073/pnas.0407817101	Proceedings of the national academy of sciences	182	2,721
8	Eschenhagen T, et al.	Three-dimensional reconstitution of embryonic cardiomyocytes in a collagen matrix: a new heart muscle model system	Article	1997	10.1096/fasebj.11.8.9240969	The FASEB journal	162	2,455
9	Shimizu T, et al.	Fabrication of pulsatile cardiac tissue grafts using a novel 3-dimensional cell sheet manipulation technique and temperature-responsive cell culture surfaces	Article	2002	10.1161/hh0302.105722	Circulation Research	160	2,515
10	Leor J, et al.	Bioengineered cardiac grafts: A new approach to repair the infarcted myocardium?	Article	2000	10.1161/circ.102.suppl_3.III-56	Circulation	156	2,399

The first important research paper in this area was a review in *Science* by Langer et al. titled “Tissue Engineering”, which was published in 1993. This review laid the foundations in tissue engineering and addressed potential challenges and applications in tissue regeneration and repair. Another highly cited paper was published in *Nature Medicine* by Zimmermann et al., in 2006, and was titled “Engineered Heart Tissue Grafts Improve Systolic and Diastolic Function in Infarcted Rat Hearts”. This study demonstrated the construction of large contractile cardiac tissue grafts *in vitro* and confirmed their viability after implantation and their ability to enhance the contractile function of infarcted hearts. In 2008, Ott et al. published a groundbreaking paper in *Nature Medicine* titled “Perfusion-Decellularized Matrix: Using Nature's Platform to Engineer a Bioartificial Heart”. This was the third-highest cited paper in this field. This study introduced the concept of perfusion decellularization for the production of a complex and biocompatible cardiac extracellular matrix scaffold, which was characterized by a perfusable vascular architecture, competent acellular valves, and an intact four-chamber geometry that provided a biomimetic template for tissue engineering (Table 5).

### Keywords analysis

We analyzed 6,970 keywords extracted from 2,273 papers using the VOSviewer but excluded search terms, synonyms, and duplicates (e.g., tissue engineering, heart, cardiac tissue engineering, cardiac tissue, heart tissue, and engineered heart tissue). The top five keywords were in-vitro (474 times), scaffolds (295 times), extracellular-matrix (277 times), mesenchymal stem cells (266 times), and cardiomyocytes (223 times) (Table 6).

**Table 6:** Top 20 keywords in the studies of CTE.

Rank	Keyword	Occurrences	Total link strength
1	*In vitro*	474	2,287
2	Scaffolds	295	1,472
3	Extracellular-matrix	277	1,389
4	Mesenchymal stem cells	266	1,280
5	Cardiomyocytes	223	1,085
6	Differentiation	216	1,126
7	Stem cells	214	1,039
8	Cells	200	862
9	Collagen	186	959
10	Biomaterials	185	934
11	Mechanical-properties	184	892
12	Transplantation	177	887
13	Scaffold	168	914
14	Heart-valves	160	703
15	Model	151	653
16	Fabrication	147	749
17	Endothelial-cells	143	656
18	Myocardial-infarction	139	740
19	Regenerative medicine	135	636
20	Matrix	133	655

**Figure 6:**
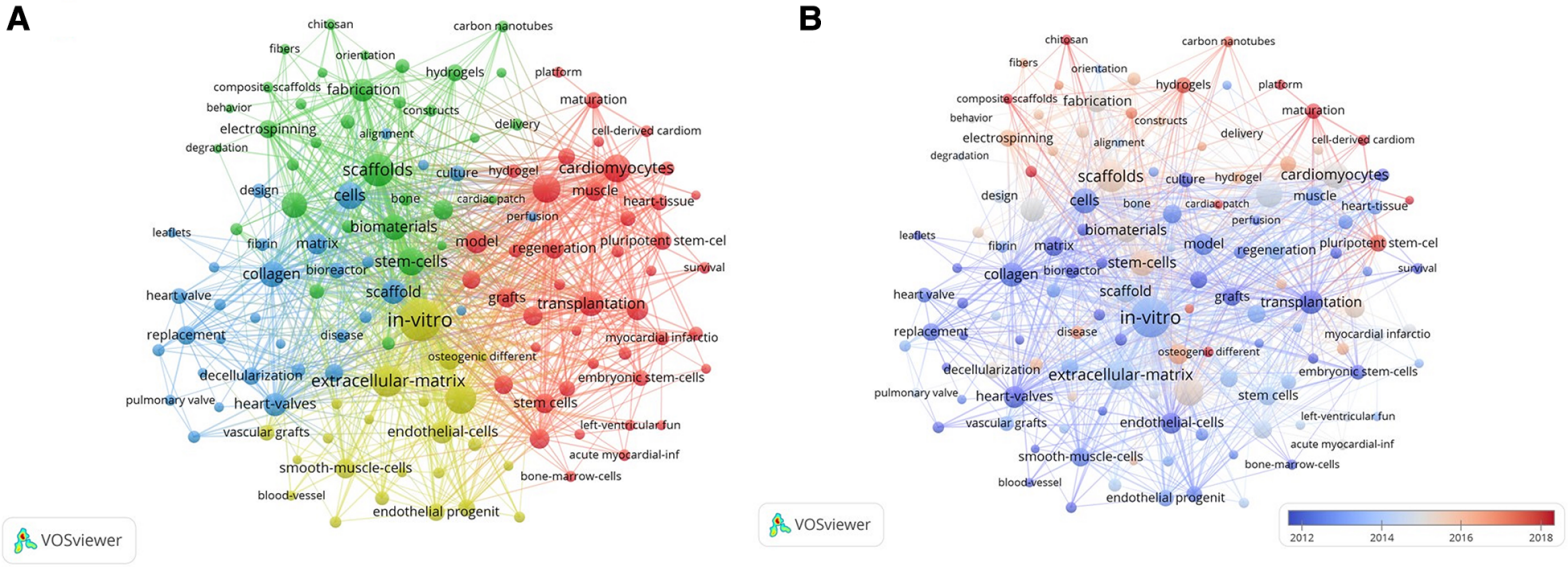
(**A**) Network map of high-frequency keywords that occur more than 30 times. A larger node size denotes a higher frequency of keyword occurrence. Different colors represent different clusters, and the lines between different nodes stand for the relationship between different keywords; (**B**) A visual map of high-frequency keywords that occurred more than 30 times over time. The node size denotes the frequency of the keyword with a larger node size representing a higher frequency. The average time of occurrence of the keywords is indicated by different colors with the blue color denoting older keywords, and red denoting the newer keywords. The relationship between different keywords is denoted by lines between different nodes.

The most frequent keywords (≥30 times) were categorized into four clusters that represented four different directions in the CTE research area (Figure 6A). The most frequent keywords in the red cluster included cardiomyocytes (223 times), differentiation (216 times), transplantation (177 times), model (151 times), myocardial infarction (139 times), progenitor cells (118 times), and regeneration (116 times).

The most frequent keywords in the green cluster included scaffolds (295 times), stem cells (214 times), biomaterials (185 times), mechanical properties (184 times), and fabrication (147 times).

The main research topics in the blue cluster were cells (200 times), collagen (186 times), scaffold (168 times), heart valves (160 times), matrix (133 times), replacement (105 times), and extracellular matrix (101 times).

The main keywords in the yellow cluster were in-vitro (474 times), extracellular matrix (277 times), mesenchymal stem cells (266 times), endothelial cells (143 times), regenerative medicine (135 times), and smooth muscle cells (114 times). Furthermore, significant overlap was observed between the four clusters, thereby indicating high complementation between various aspects of research studies related to CTE.

The average published year (APY) indicated the novelty of the keywords. The keywords with a high APY index included functional maturation (red cluster, APY: 2018.63, 30 times), cell-derived cardiomyocytes (red cluster, APY: 2018.43, 46 times), maturation (red cluster, APY: 2018.17, 84 times), composite scaffolds (green cluster, APY: 2018.54, 41 times), patch (blue cluster, APY: 2017.34, 32 times), and osteogenic differentiation (yellow cluster, APY: 2018.32, 50 times). These keywords are considered potential hotspots in this research area.

### Combined evolutionary path

Early keywords (2012 to 2014) in this research area included in-vitro, collagen, and transplantation; the emerging keywords were maturation, hydrogels, platform, multipotent stem cells, and cell-derived cardiomyocytes, which showed strong association with the other keywords and potentially represent current study frontiers in CTE (Figure 6B).

**Figure 7:**
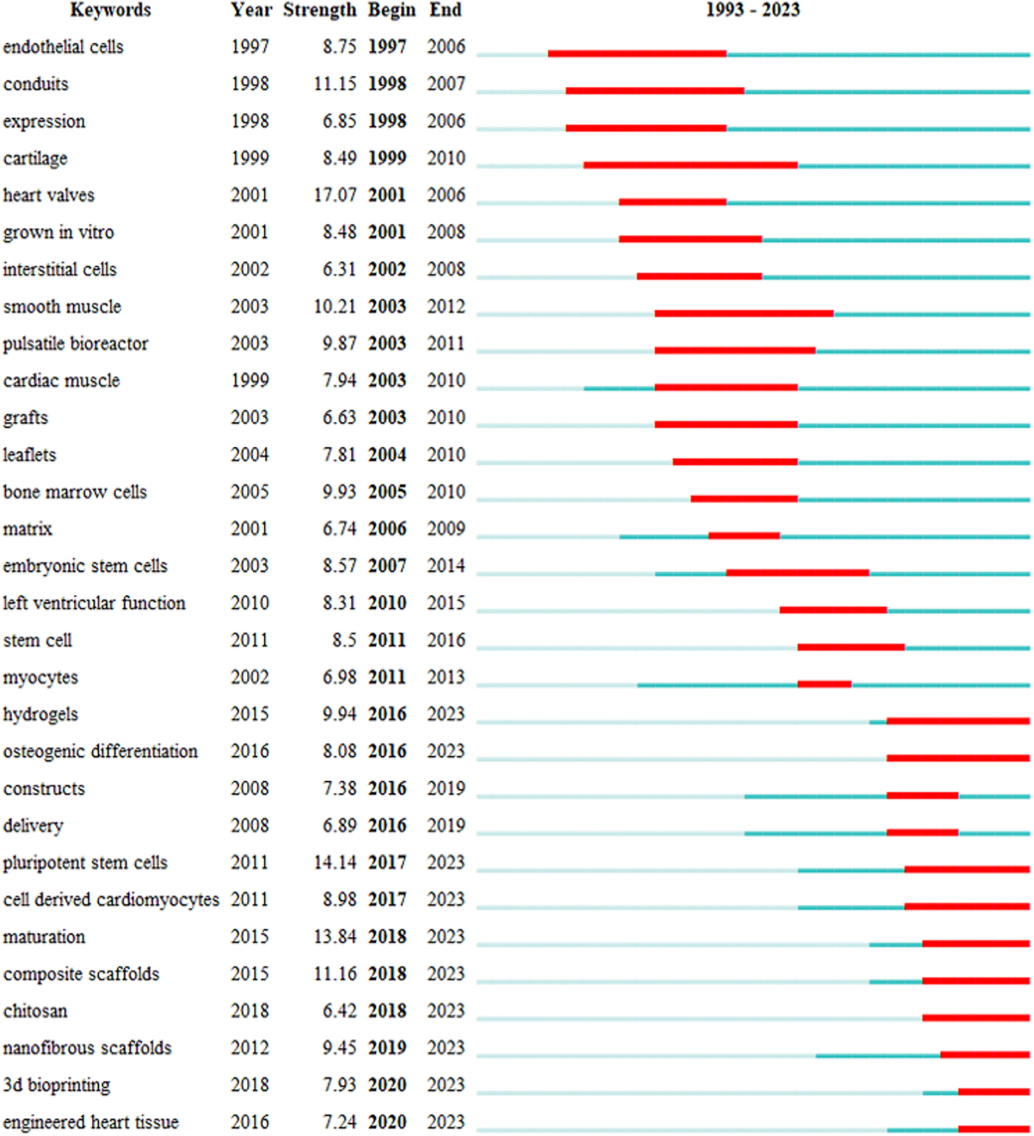
The top 30 keywords with the strongest citation burst.

The top 35 keywords with high burst intensities and the burst year were generated by CiteSpace. The main emerging keywords with a high citation strength in the field of CTE were pluripotent stem cells (citation strength: 14.14, 2017–2023), maturation (citation strength: 13,84, 2018–2023), composite scaffolds (citation strength: 11.16, 2018–2023), hydrogels (citation strength: 9.94, 2016–2023), and nanofibrous scaffolds (citation strength: 9.45, 2019–2023) (Figure 7).

## Discussion

To the best of our knowledge, this study represents the first bibliometric analysis conducted on CTE. This analysis resulted in some key findings. First, CTE studies have increased over time. Second, CTE studies involve collaborative research between diverse scientific disciplines. Third, studies originating from the United States and Europe have significantly influenced the research findings on CTE so far. Moreover, China has emerged as a prominent contributor in recent years with a significantly higher number of publications among emerging countries. Lastly, functional maturation of stem cells and the application of biomaterials research are potential research hotspots in this area.

CTE offers a promising therapeutic strategy for patients with myocardial infarction and ischemic cardiomyopathy, especially in cases showing limited differentiation of the injected stem cells into cardiomyocytes even after reaching the injury site (29). There has been a significant upward trend in CTE-related research over the last three decades as indicated by 165 publications in 2021 compared to a single publication in 1992. There have been several seminal research papers in this area. In 1993, a review by Langer et al. in *Science* summarized the foundations and challenges of this interdisciplinary field and addressed the quest for solutions in the area of tissue regeneration and repair (30). Subsequent studies have contributed significantly to progress in CTE through novel findings and innovations. In 1997, Eschenhagen T et al. reported the generation of a novel heart muscle model system based on the three-dimensional reconstitution of embryonic cardiomyocytes (31). Additionally, Niklason L et al. showed *in vitro* growth of functional arteries using tissue engineering approaches (32). These pioneering studies laid the groundwork for further advancements in CTE. The use of grafts (33) and bioengineered scaffold (34) has further propelled rapid progress in the field.

Similar to other research areas (35, 36), the United States emerged as a dominant leader in CTE research, with 916 publications. This can be attributed to the following three factors: (1) Early research in CTE was initiated in the United States and a solid foundation was established for subsequent progress; (2) There has been significant emphasis on advancing CTE research in the United States as is evident from the notable contributions of authors such as Vunjak-Novakovic G (37), Shinoka T (38), Breuer CK (39), and Mayer JE (40); and (3) the United States has invested significantly in engineered heart tissue (EHT) research as evidenced by ∼700 publications in this field being supported by US-based funding sources. This financial support has fueled the progress and innovation in CTE research within the United States.

China is an emerging country in the field of CTE research, as evidenced by 223 publications between 1992 and 2021. The National Natural Science Foundation of China ranked fourth among the top ten funding organizations. This highlighted China's commitment to CTE research. Chinese researchers have primarily focused on several hot topics within the field, including the application of scaffolds (41), hydrogel (42), nanomaterial (43), and the development of injectable and conductive cardiac patches (44). These trends suggest that China will contribute significantly to the field of CTE in the future and the number of citations for publications from China is expected to increase over time.

The United States is the most influential country in the field of CTE because research papers from the USA have the highest number of citations (52,377 times). The average citation rate of papers from the United States is 57.18 times/paper. This influence is attributed to a number of high-quality studies conducted by American researchers. Moreover, several highly cited papers in the field of CTE, including “Perfusion-decellularized matrix: using nature's platform to engineer a bioartificial heart” (45), *“*Functional living trileaflet heart valves grown in vitro” (46), *“*Functional arteries grown in vitro*”* (32), and “Functional assembly of engineered myocardium by electrical stimulation of cardiac myocytes cultured on scaffolds*”* (34), were authored by researchers from the United States. Furthermore, four of the top ten influential institutions in the research area of CTE are from the United States. This further reiterates the high impact of the United States in this field. Researchers from Harvard University focused primarily on the three-dimensional bioprinting of engineered tissue (47) and engineered three-layer scaffolds (48). Researchers from the Massachusetts Institute of Technology (MIT) focused on the application of hydrogels (49) and nano-biomaterials (50) in EHT. Researchers from the Pennsylvania Commonwealth System of Higher Education specialized in the area of musculoskeletal tissue repair (51). Researchers from the University of California focused on the application of injectable microporous gel scaffolds in tissue engineering (52) and drugs related to the biological function of the engineered tissue (53). Our data indicated that researchers from the United States established extensive collaborations with researchers from Canada, France, and England. Cooperation between different countries and institutions was instrumental in the advances made in this field.

Multidisciplinary investigations are common in studies related to CTE and have played a pivotal role in advancing the field. CTE research encompasses a wide range of disciplines, including biochemistry, molecular biology, materials science, and cell biology. Research scholars have actively investigated various areas within these disciplines for the application of siliceous sponges (54)and virus-based scaffolds (55) in engineering cardiac tissues and gaining a deeper understanding of the processes involved in tissue transplantation (56). The development of these related disciplines has significantly advanced CTE research, fostered collaboration, and driven the progress of this field.

Keywords are words or phrases that represent the main content and ideas represented in the paper and indicate the research focus and direction. The high-frequency keywords were “*in vitro*” (474 times), “scaffolds” (295 times), and “extracellular-matrix” (277 times), which highlighted the main research areas in CTE. The occurrence of keywords changes over time and the emerging keywords in top journals signify the potential research directions in the field. For example, emerging keywords such as “functional maturation” (57, 58), “platform” (59), “scaffold” (60), “hydrogel” (61), and “cell-derived cardiomyocytes” (62) were not listed in the top 20 most frequently mentioned keywords but demonstrate potential research directions. This aligns with those keywords with higher average published year and strength, which further indicates those are the potential future research directions in CTE.

## Conclusion

In summary, the CTE technique shows significant promise in treatment for patients with cardiovascular diseases, including myocardial infarction and ischemic cardiomyopathy. Since 2011, publication rates in the field of CTE have increased significantly, thereby suggesting its status as a hot research topic. The United States is a dominant player in CTE research with a significantly high number of publications and citations compared to other countries. China is emerging as a significant contributor in the field. Interdisciplinary collaboration, especially between engineering and material sciences, has played a critical role in the progress of CTE. The future directions of research in CTE include the functional maturation of stem cells and the development of novel biologically relevant materials and related applications.

## Data Availability

The original contributions presented in the study are included in the article/Supplementary Material, further inquiries can be directed to the corresponding author/s.
